# Alkaloid Accumulation and Distribution within the Capsules of Two Opium Poppy (*Papaver somniferum* L.) Varieties

**DOI:** 10.3390/plants13121640

**Published:** 2024-06-14

**Authors:** Péter Májer, Éva Zámboriné Németh

**Affiliations:** Department of Medicinal and Aromatic Plants, Institute of Horticultural Sciences, Hungarian University of Agriculture and Life Sciences, 1118 Budapest, Hungary

**Keywords:** alkaloids, morphine, poppy, *Papaver somniferum*

## Abstract

The goal of this study was to clarify the role of capsule size and morphology in the alkaloid yield of poppy. In 2023, two industrial varieties from large-scale cultivation were investigated. Three classes of capsule size (large, medium, and small) and four organelles (wall, placenta, disc, and thalamus) of the seedless capsule were studied for their mass proportions and alkaloid contents. In ‘Meara’, large capsules had 41% lower total alkaloid levels than smaller ones. In ‘Morgana’, there was no difference in total alkaloids between size groups, but large capsules had higher contents of codeine and thebaine. Among the four organelles, the wall represented the largest mass in both varieties (60–67%), while the disc and the thalamus gave the lowest proportions (below 9%). In the variety ’Meara’, the highest alkaloid contents appeared in the wall (2.69% d.w.), followed by the placenta, and the other two parts. ‘Morgana’ accumulated the highest alkaloid content (3.72% d.w.) in the placenta. Morphine follows the trend of the total content, while codeine and thebaine may differ. Accurate information on the accumulation of alkaloids in the generative organs may contribute to increasing effectiveness in target-oriented breeding and optimization of cultivation with an appropriate choice of variety.

## 1. Introduction

Opium poppy (*Papaver somniferum* L.) is one of the most ancient cultivated plants [1]. Historically, people have produced it primarily for opiate alkaloids; however, today, the utilization of the seeds and their valuable fatty oil is also popular and showing a growing market [2]. In the most important poppy-producing countries, cultivation is regulated and culinary varieties with low alkaloid content are grown separately from industrial varieties with high alkaloid contents [3]. The most important alkaloids of poppy are the morphinanes: morphine, as a strong antinociceptive and sedative agent; codeine, used mostly against spasmodic cough; and thebaine, which today is the starting compound of semi-synthetic opioids like oxymorphone and hydrocodone [4]. The current pharmaceutical industry usually extracts these alkaloids from the dry, seedless capsules called poppy straw.

The quality of the poppy straw depends on its alkaloid concentration and composition [5]. The yield of the alkaloids per unit field area is determined by both the alkaloid ratio and the yield of the capsules. Therefore, in crop production, both factors should be optimized, especially under the challenges of climate extremes.

The efficacy of the cultivation may be increased with different tools. Kara and Bajdar [6] described a beneficial effect of early sowing on the alkaloid content of poppy capsules in Turkey. A later sowing had negative effects on the biomass too [7]. Due to heavy rains, a significant ratio of the alkaloids may be extracted from the capsules, and thus, lost; moreover, the alkaloid spectrum might also change because of differences in the water solubility of the compounds [8,9]. Extraction loss is highest in the case of the capsule wall, while the placenta is less threatened [10].

However, the principal factor for a high alkaloid production is the genotype (variety), whose genetic potential could be realized in the cultivation. The alkaloid content of industrial varieties has notably been elevated in recent decades. The data from the study by Fist [11] allow one to conclude that, in the 1990s, in the countries with intensive poppy cultivation (Australia, France, and Spain), the mean alkaloid content of poppy straw was 0.87–1.82%. Around the same period, the first varieties with stable alkaloid contents above 1% were also introduced into cultivation in Central Europe. In the last 20 years, alkaloid content has grown further, as a result of breeding. The best varieties in Europe may provide 1.5–2.5% morphine content even under field conditions [12,13,14]. In Australia, the average morphine content of the crop is 1–3% [15]. According to published data, in Turkish varieties, the alkaloid concentration seems to be lower; their highest values were 1.14% [16] and 1.61% [17] in the last decade. In Slovakia and the Czech Republic, poppy varieties have been developed for double uses (pharmaceutical and culinary) with moderate alkaloid contents (0.3–1.0%) [18,19].

The available data generally refer to the harvested poppy straw, but the alkaloid concentration in each variety also depends on the ratio of different plant parts. In industrial practice, it is widely known that a higher ratio of stem in the harvested material may seriously deteriorate the quality. By cutting the stemless capsules, the alkaloid content of the poppy straw may be elevated by approximately 30%.

Nevertheless, data about the distribution and concentration of alkaloids inside the capsule are rare in the literature. Nash [20] evaluated poppy capsules originating from Tasmania and established that terminal and lateral capsules provide significantly uniform alkaloid concentrations. In both types of capsules, the total alkaloid content was highest in the wall (1.5–1.7% d.w.) followed by the placenta (1.4% d.w.), while in the disc, it was much lower. Additionally, Bernáth [21], in examining an Australian genotype in Hungarian cultivation, also measured higher values in the capsule wall (1.7–2.3%) and lower ones in the placenta (1.0–1.4%).

The connection of capsule size and its alkaloid production has also been rarely studied. Harvest et al. [22] demonstrated a positive linear correlation between capsule dry mass and capsule morphine mass but suggested no connection between capsule mass and the concentration of morphine. Kálmán-Pál et al. [23] explained that the correlation between morphine content (% d.w.) and capsule mass (g/plant) may vary according to genotype and environmental conditions.

These questions are even more complex considering that poppy has a very large morphological variability in both vegetative and generative organs. In the UPOV (International Union for the Protection of New Varieties of Plants) system, the test for the evaluation of poppy varieties includes 32 different characteristics, among which 11 are determined for the capsule. Five capsule forms and five disc forms are distinguished. However, in practice, several other transitive forms can be observed too [24].

If the organic localization of the alkaloids is known, altering their ratio may also be a tool in enhancing the alkaloid production in new varieties. Therefore, we examined two recently developed, high alkaloid-containing Central European varieties to detect the alkaloid accumulation in capsules of different sizes and in their separated parts. More precise knowledge of the accumulation of alkaloids in the generative organs may contribute to increasing the effectiveness of target-oriented breeding and to improving the efficiency of cultivation with an appropriate choice of variety.

## 2. Results

**Figure 1:**
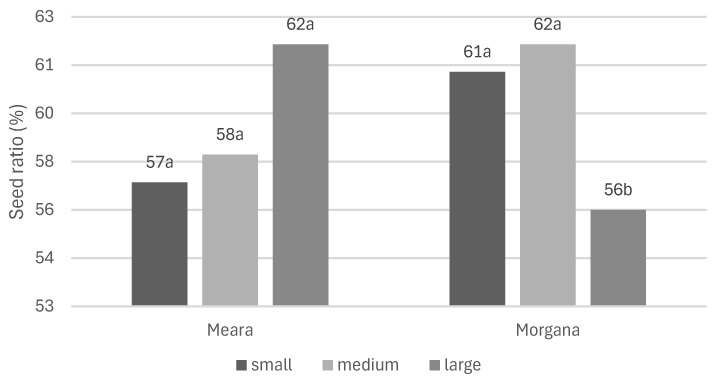
The seed ratio in total mass of capsule (m/m%). Data were calculated as mass of seeds/(mass of seeds + mass of empty capsules). Values within columns of the same variety with the same letters (a, b) are not significantly different (*p* < 0.01).

In the ‘Meara’ variety, the large capsules contained a higher ratio of seeds (62%) than the medium (58%) and small (57%) capsules, but without significant differences to each other (Figure 1). The alkaloid content of the large capsules is significantly (*p* < 0.001) lower than that of the medium- and small-sized capsules (2.36 and 2.38%, respectively). The main alkaloid, morphine, shows the same tendency: 2.28% d.w. was detected in both the small- and medium-sized capsules, while only one-third of that was measured in the large ones. The codeine content of the capsules was very low (0.07–0.10% d.w.) without significant differences among the fractions, and thebaine could not be detected in the samples (Table 1).

In the case of the ‘Morgana’ variety, the ratio of the seeds was significantly (*p* < 0.01) lower (56%) in the large capsules than in the medium- (62%) and small-sized (61%) ones (Figure 1). However, concerning both total alkaloid content (2.43–2.71%) and morphine concentration (2.31–2.41%), we did not detect significant differences among the three groups of capsules. At the same time, larger capsules were able to accumulate significantly higher concentrations of both codeine (0.21%, *p* < 0.01) and thebaine (0.15%, *p* < 0.001). Smaller capsules contained only one-third of codeine and 5–10 times less thebaine than these values.

**Figure 2:**
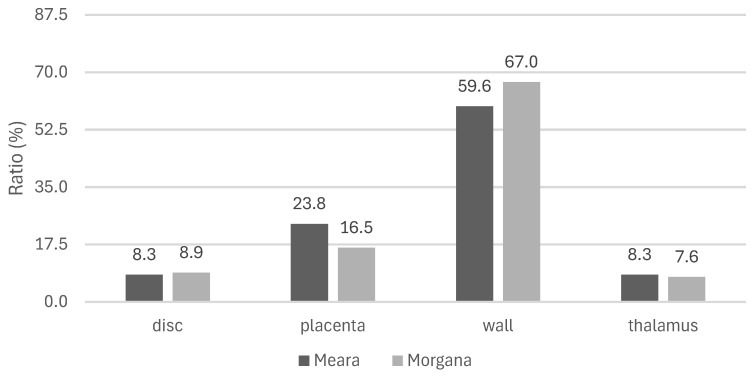
Ratio of capsule parts in the total mass of capsules in the experimental varieties.

As for the seedless capsule, in the variety ‘Meara’, we measured a ratio of 8.3% for both the disc and thalamus compared to the total seedless capsule mass. The wall has the highest proportion (59.6% as a mean) within the studied medium-sized capsules, and the placenta has the second largest ratio with 23.8%. In the other variety, the capsule wall provides an even higher ratio of the total seedless mass (67%), the placenta has a mean of 16.5%, and the disc and the thalamus give 8.9% and 7.6%, respectively (Figure 2).

The different parts of the seedless capsules showed variable alkaloid contents (Table 2). In ‘Meara’, the total alkaloid content of the thalamus and that of the disc was 0.69% and 1.02%, respectively, with no statistical difference between them. A significantly (*p* < 0.001) higher value was measured in the placenta (2.13%), and an even higher content in the wall (2.69%). The tendency is the same for the ratio of morphine in the different parts of the capsule. Thebaine and codeine are very low in this variety; a significantly higher accumulation was only detected in the wall for codeine (0.17%).

In ‘Morgana’, the lowest total alkaloid content was found in the thalamus (0.76%) and in the disc (0.92). In the wall, the content was significantly (*p* < 0.001) higher (2.76%); however, the highest concentration could be found in the placenta (3.72%). As for the morphine ratio, the examined capsule parts show a similar order: the lowest and statistically identical contents in the disc and thalamus, a higher value (2.5%) in the wall, and the highest one (3.34%) in the placenta. In this variety, the other morphinanes also demonstrated significant differences: both for the codeine and thebaine, the placenta accumulated the highest concentrations.

Examining the data of the organs, we can state that the variety and the organ significantly influence the development of morphine, codeine, thebaine, and the total alkaloid content in the samples. The effect of the variety–organ interaction is also significant for each of these characteristics (Table 3).

**Figure 3:**
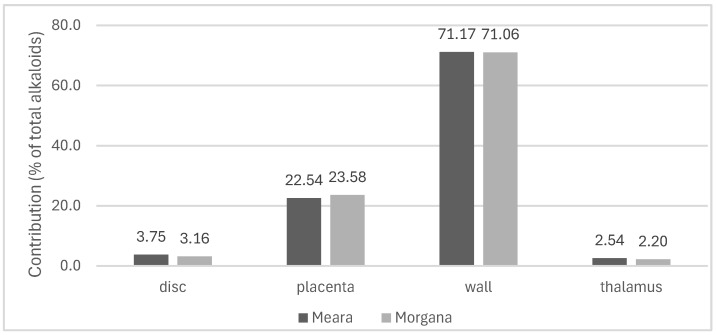
Contribution of the different parts of seedless capsules to the total alkaloid yield of a single capsule. Data were calculated as the mass of seedless capsule × ratio of capsule part × alkaloid percentage.

Calculating the contribution of the different parts of seedless capsules to the total alkaloid yield of a single capsule, we established that the wall accounts for 71%, while the placenta accounts for 23–24% of the production. The difference between varieties is negligible (Figure 3).

## 3. Discussion

The ratio of the seeds compared to the seedless parts of the capsules proved to be different in the large, medium-sized, and small capsules. In both varieties, the large-sized capsules showed different values compared to the medium and small ones; however, this is the opposite in the two genotypes.

Data about these values are scarce in the literature. Nash [20] determined a 53–56% seed ratio/capsule total dry matter but without mentioning capsule size. According to Singh et al. [25], there is a strong correlation between seed yield and capsule size. Bernáth [21], however, declared that the correlation between capsule weight and seed weight is not constant, but depends on weather conditions and genotype. We have to add that they measured individual yields of plants and not ratios/correlations based on individual capsule data; therefore, the number of capsules/plants might also have influenced the calculated values. Based on our present data, we can declare that capsule size significantly influences the ratio of seeds in the capsules; however, other factors like variety or environmental conditions may also affect the exact values. Obviously, in bigger capsules, the placenta may also be bigger, and thus, more ovaries will be present. To the best of our knowledge, no detailed investigations have been carried out about the quantity and quality of seeds in connection with their place on the placenta or its size. However, it is clear that, for the development of viable seeds, a successful fructification is also necessary, which might be influenced by the genetic background or even by weather conditions. Based on our experience (not published), the pollen tube is sometimes not able to reach the ovaries around the bottom of large capsules, especially if they have an elongated form. Hot and dry weather during blossoming may contribute to a bad fructification [10]. Therefore, in breeding, large capsules alone do not necessarily lead to higher yields either of seeds or of alkaloids. During variety development, all of the above questions should be studied carefully.

The yield characteristics and the alkaloid accumulation of the different size capsules were different in the two tested varieties. While large capsules of ‘Meara’ showed a lower content than the smaller ones, there was no statistical difference among either group of capsules in ‘Morgana’. At the same time, significantly higher contents of codeine and thebaine were measured in larger capsules of the latter variety compared to the small and medium ones. Fairbairn and Kapoor [26] measured the distribution and diameter of the laticifers in different sized capsules, and according to this histological study, there was no significant difference among them.

They mentioned that the development of laticifers in each capsule is similar, and they reach the maximum diameter two weeks after petal fall. The authors added that, as a result, the capsule reaches its maximum yield of latex at that time. Although they theorized that the peak in latex yield also means a peak in alkaloid yield, this was challenged by Harvest et al. [22] who measured a continuous increase in morphine concentration (% d.w.) until full ripeness. As in their trial, latex capacity (maximum ratio of laticifers calculated to capsule mass) was already reached one week after petal fall, they concluded that the alkaloid concentration was not closely connected with the mass of latex, but more likely with the number and size of vesicles in it. The mentioned increase in alkaloid content during capsule development coincides with the findings of Kálmán-Pál et al. [23], although they did not investigate the anatomical structure of the capsules.

It can be established that neither of the above-cited authors evaluated the morphine concentration in connection with capsule size or with the ratio of its seedless parts. According to Harvest et al. [22], there was a positive linear correlation between capsule dry matter, capsule morphine mass, and latex mass. By this, they supported the results of Singh et al. [25], which showed that larger capsules have a higher morphine yield due to their greater mass of latex. Nevertheless, as the morphine concentration does not seem to be directly linked to latex mass, the cited data may be valid only in certain cases in single investigated varieties, and cannot be generalized. Thus, they do not give any information about the topic studied by us: the connection between capsule mass and the concentration of individual alkaloid compounds.

Based on an experiment with two varieties under controlled conditions, Kálmán-Pál et al. [23] demonstrated that no constant correlation can be established between capsule mass and the total concentration (% d.w.) of morphinane alkaloids. However, this calculation was made with capsule mass per plant and not with that of the sampled individual capsules, and therefore, it may not be exactly relevant for our study either.

The proportion of the four parts of the seedless capsule gave slightly different values in the two studied varieties, although in both of them, the wall represented most of the mass: 60% and 67% in ’Meara’ and ’Morgana’, respectively. The disc and the thalamus gave the lowest proportions in each case (7.6–8.9%). Formerly, only Nash [20] studied this question in a no-name Australian genotype and stated too that the wall provided the highest mass ratio (54%). However, their measurements included some stem parts as well.

The different parts of the seedless capsules showed characteristic differences in their alkaloid contents, but differences between the two tested varieties were also detected. The variety ‘Meara’ followed a similar accumulation pattern to that mentioned in the literature [10,20]: the highest contents in the wall, followed by the placenta, and the lowest ones in the other two capsule parts. In these mentioned studies, only single varieties were examined. However, in our trial, the other variety, ‘Morgana’, showed another characteristic of having the highest alkaloid contents in the placenta and significantly lower values in each of the other capsule parts. With this, we demonstrated how placentas have a greater role in the morphine yield of the capsules than was formerly mentioned by Nash [20]. A variety of characteristics are shown also by the fact that, in ‘Meara’, there is a 3.9-fold difference between the capsule parts of the highest and lowest alkaloid contents, while in ‘Morgana’, this difference is 4.9-fold.

From this study, it was also established that only the major alkaloid compound morphine follows the tendency of the total contents, while the concentrations of codeine and thebaine may be somewhat different from that. This might be in connection with the differences in laticifer and phloem structure in the four mentioned capsule parts [26] as the biosynthesis of morphinanes is characteristically divided between these two cell types [27]. While for thebaine, the synthesis occurs in the phloem part, further enzymatic transformation processes mostly take place in the laticifers. The detection of these structural differences in different capsule parts and poppy varieties requires further anatomical studies.

## 4. Materials and Methods

The object of these investigations were two Slovakian industrial poppy (*Papaver somniferum* L.) varieties ‘Meara’ and ‘Morgana’, cultivated in large-scale fields in Hungary. The two poppy fields were located near to each other (within 10 km distance), and the ecological characteristics of the fields were similar. During the vegetation time of the poppy, the mean temperature was 14.0 °C and the amount of precipitation was 269 mm. The area had meadow-alluvial soil, rich in phosphorous and potassium. To ensure the proper amount of nitrogen, 250 kg/ha lime ammonium nitrate was used. The sowing of the poppy was carried out at the end of February (‘Morgana’) and beginning of March (‘Meara’). The row distance was 12 cm, and seed dosage was 2 kg/ha. Weed control was conducted in 4 leaf stages with mesotrione and in 8 leaf stages with tembotrione; furthermore, insecticide treatment was applied after flowering (lambda-cihalotrin).

Capsule samples were collected in full ripening phase in July 2023. We examined the healthy terminal capsules without any stem part.

**Figure 4:**
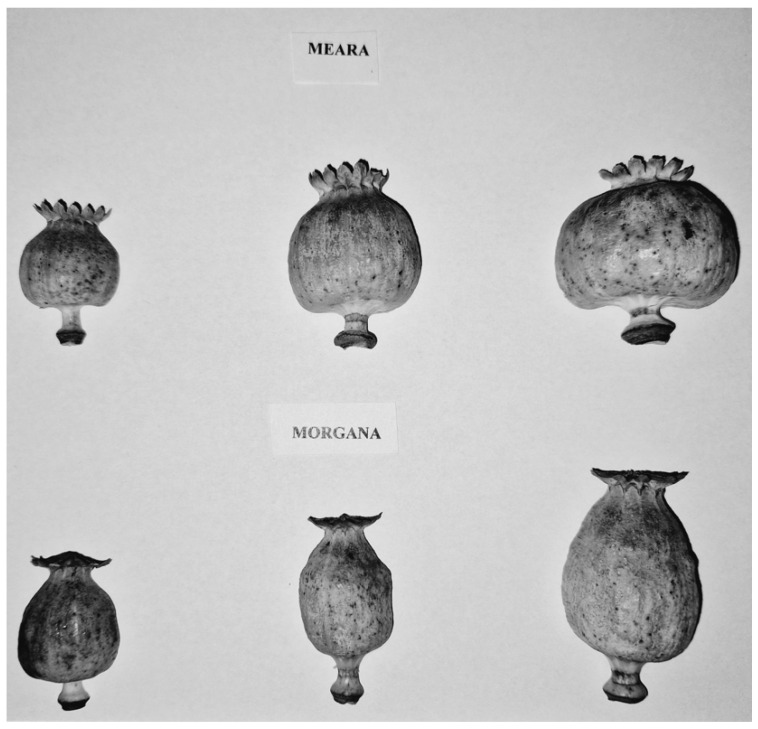
Capsules of different sizes in ‘Meara’ and ‘Morgana’ varieties. Small-, medium-, and large-sized capsules from left to right (see also Table 4).

We grouped the capsules according to their sizes: large, medium, and small ones (Figure 4, Table 4). The capsules were separated into seed and seedless parts, and their masses were determined by the accuracy of 10^−2^ g. Sixteen replicates were measured from both varieties and each capsule size group.

**Figure 5:**
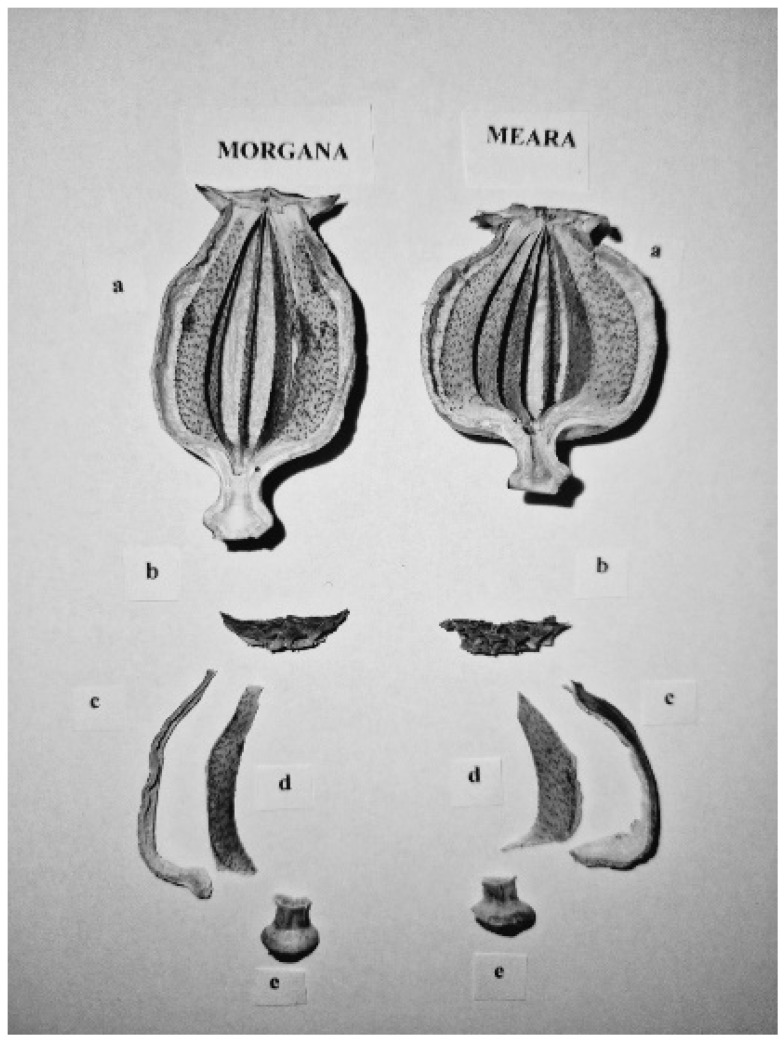
Capsules and capsule parts of the experimental varieties (a: seedless whole capsule; b: disc; c: wall; d: placenta; e: thalamus).

To determine further mass proportions and alkaloid concentrations of the capsules, we used the “medium” size group and separated 16 capsules from each variety into the following fractions: stigmatic disc, wall (pericarp), placenta, and thalamus (receptacle) (Figure 5). We determined the mass of each capsule part separately.

We ground and homogenized the samples using a rotary blade grinder to a fine powder (particle size < 0.2 mm) for the quantitative analysis of alkaloids. For the preparation of a sample, we used 4 seedless capsules or organelles. From each sample, we took 200 mg of powder, which we then extracted with 50 mL of extraction solvent (50% methanol, 44.8% water, and 5.2% formic acid). We used an ultra-high-performance liquid chromatography instrument (Dionex Ultimate 3000 RS, Thermo Scientific). It was connected to a high-resolution, accurate-mass (HRAM) mass spectrometer (Q Exactive Orbitrap, Thermo Scientific), which was equipped with electrospray ionization (ESI) source. A Thermo Accucore C18 column (100 mm × 2.1 mm and 2.6 μm particle size) was used to separate the alkaloids. The manufacturer of the MS and UHPLC equipments is Thermo Fisher Scientific Inc. (Waltham, MA, USA). The column was thermostated to 25 °C (±1 °C). The isocratic method (7:3 mixture of methanol (B) and water (A), both acidified with 0.1% formic acid) was used for the chromatographic separation. The solvent flow rate and injection volume was set at 0.2 mL/min and 2 μL, respectively. Run time: 12 min. ESI spectra were recorded in positive ion mode using selective ion monitoring at a resolution of 70,000. The scan range was set from 100 to 500 *m*/*z*. The internal standard (Morphine-d3) method was used for the quantitative determinations with a 7-point calibration curve (concentrations: 1, 10, 25, 50, 75, 100, and 150 ng/mL). The obtained data were processed with Thermo Scientific Xcalibur 3.1 software. Contents of morphinanes (morphine, codeine, and thebaine) and their sums were calculated with reference to the dry matter. The measurement of the alkaloid concentrations was carried out in 4 replicates.

Statistical analysis was performed using the IBM SPSS Statistics 29.0.1.0 program and one-way ANOVA for each variable in both cultivars. Significant differences among samples were determined using the Tukey post hoc test. Homogeneity of variances was tested by Levene’s method or Welch’s test. For the data of the organs, we also used a two-way ANOVA to explore the influence of the variety and the organ, as well as the interaction.

## 5. Conclusions

Our hypothesis about the role of capsule size and genotype in the seed ratio and alkaloid accumulation of poppy has been confirmed. Bigger capsules may not necessarily guarantee proportionally more seeds than smaller ones, as the seed ratio also depends on the genetic background of the variety and presumably on other factors (capsule form and weather conditions).

In large capsules, the alkaloid content (% d.w.) and the ratio of morphinanes (morphine, codeine, and thebaine) may be different from the content in smaller ones, but the actual concentrations depend on genotype.

In both of the examined European varieties, the mass proportion of the organs inside the seedless capsule was similar to the largest ratios for the wall, followed by the placenta, and the lowest masses—similar to each other—exhibited in the disc and the thalamus.

Our data showed significantly higher accumulation levels of morphine and total alkaloids in the capsule wall and the placenta compared to the disc and the thalamus. However, the difference between wall and placenta seems to be variety dependent and also not necessarily the same for thebaine and codeine. In practice, varieties accumulating the highest level of alkaloids (especially morphine) in the placenta may be favorable, as they are at less of a risk of being washed out by the rain before harvest.

More precise knowledge of the accumulation of alkaloids in the generative organs may contribute to increasing the effectiveness of target-oriented breeding and to improving the efficiency of cultivation with an appropriate choice of variety.

## Figures and Tables

**Table 1:** Size and mass characteristics and alkaloid contents of the capsules of the experimental varieties.

Variety	Meara			Morgana		
Fraction	Small	Medium	Large	Small	Medium	Large
Height (mm) *	36.13 a	44.56 b	55.13 c	37.63 a	42.50 b	53.88 c
Width (mm) *	24.44 a	33.63 b	42.44 c	19.81 a	25.19 b	34.94 c
Seeds (g/capsule) *	1.54 a	3.32 b	6.13 c	1.40 a	2.57 b	4.68 c
Empty capsule (g) *	1.16 a	2.38 b	3.82 c	0.91 a	1.58 b	3.68 c
Morphine (m/m%) *	2.28 a	2.28 a	0.90 b	2.41 a	2.31 a	2.35 a
Codeine (m/m%) **	0.08 a	0.10 a	0.07 a	0.07 a	0.10 a	0.21 b
Thebaine (m/m%) *	0.00 a	0.00 a	0.00 a	0.01 a	0.02 a	0.15 b
Sum of alkaloids (m/m%) *	2.36 a	2.38 a	0.97 b	2.49 a	2.43 a	2.71 a

All values are means of 16 replicates for size and mass traits and 3 replicates for alkaloids. Values within rows with the same letters (a, b, c) are not significantly different (* *p* < 0.001, ** *p* < 0.01).

**Table 2:** Alkaloid content of the different parts of capsules in the case of the two varieties.

Variety	Meara				Morgana			
Organ	Disc	Placenta	Wall	Thalamus	Disc	Placenta	Wall	Thalamus
Weight (g) *	0.25 a	0.72 b	1.80 c	0.25 a	0.26 a	0.48 b	1.95 c	0.22 a
Morphine (m/m%) *	0.98 b	2.05 c	2.51 d	0.63 a	0.86 a	3.34 c	2.50 b	0.63 a
Codeine (m/m%) *	0.04 a	0.08 a	0.17 b	0.04 a	0.05 a	0.22 b	0.17 b	0.07 a
Thebaine (m/m%) **	0.00 a	0.00 a	0.01 a	0.02 a	0.01 a	0.16 b	0.09 ab	0.06 ab
Sum of alkaloids (m/m%) *	1.02 a	2.13 b	2.69 c	0.69 a	0.92 a	3.72 c	2.76 b	0.76 a

All values are means of 16 replicates for weight and 3 replicates for alkaloids. The data refer to medium-sized capsules. Values within rows with the same letters (a, b, c, d) are not significantly different (* *p* < 0.001, ** *p* < 0.01).

**Table 3:** The influence of variety and organ on the alkaloid content of samples based on two-way ANOVA.

Source of Variation		Morphine	Codeine	Thebaine	Sum of Alkaloids
Variety	F(1;24)=	24.407	24.423	30.918	33.005
	*p*=	<0.001	<0.001	<0.001	<0.001
Organ	F(3;24)=	329.201	55.662	5.684	268.305
	*p*=	<0.001	<0.001	<0.005	<0.001
Variety × Organ	F(3;24)=	32.309	13.803	6.498	31.725
	*p*=	<0.001	<0.001	<0.005	<0.001

**Table 4:** The height and width of the capsules of the two varieties.

Variety		Meara			Morgana		
Capsule		Minimum	Maximum	Mean	Minimum	Maximum	Mean
Height (mm)	small	31	40	36.13	31	40	37.63
	medium	41	50	44.56	41	50	42.50
	big	51	60	55.13	51	60	53.87
Width (mm)	small	21	30	24.44	15	24	19.81
	medium	31	40	33.63	25	30	25.19
	big	41	50	42.44	31	40	34.94
Shape index *	small	1.24	1.67	1.48	1.65	2.30	1.92
	medium	1.16	1.52	1.33	1.46	1.83	1.69
	big	1.18	1.46	1.30	1.28	1.81	1.56
Weight (g)	small	1.83	3.83	2.70	1.29	3.55	2.31
	medium	4.21	7.05	5.70	3.33	5.83	4.15
	big	7.20	13.04	9.95	6.58	10.21	8.36

* Shape index was calculated as height/width.

## Data Availability

The data presented in this study are available within the article.
